# To the Medical Profession

**Published:** 1850-11

**Authors:** Austin Flint

**Affiliations:** Buffalo, New York


### EDITORIAL DEPARTMENT.

### To the Medical Profession.

The undersigned, Chairman of the Standing Committee on *Practical Medicine,* appointed by the American Medical Association, May, 1850, respectfully solicits the co-operation of members of the Medical Profession in furnishing materials for the Annual Report in May, 1851. The duty of this Committee, as defined by the Constitution of the Association, is to "prepare an Annual Report on the more important improvements effected in this country in the management of individual diseases; and on the progress of Epidemics; referring, as occasion requires, to medical topography, and to the character of prevailing diseases in special localities, or in the United States generally, during the term of their service." In order to fulfill the objects thus expressed, the requisite data must be supplied by medical practitioners in different sections of the Union. This is more particularly true with reference to the "*progress of epidemics*" and "*the character of prevailing diseases in special localities.*" Communications, therefore, are particularly desired from persons residing in places in which Epidemics have prevailed, or in which prevailing diseases have been marked by special characters during the present year. Epidemic Cholera and Dysentery are known to have prevailed more or less extensively in different parts of the country during the past summer. Facts bearing upon the features peculiar to the present season, the production, diffusion, mortality, treatment, etc., of these diseases, will be acceptable. It is requested that Communications upon these or any of the subjects coming under the cognizance of the Committee, be transmitted to the undersigned by the first of March, 1851.

All contributions with which the Committee may be favored, will receive due attention and acknowledgment.

AUSTIN FLINT.

Buffalo, New York, Nov., 1850.

Editors of Medical Journals will confer a favor by copying the above.

